# No effects of high- *v*. low-protein breakfast on body composition and cardiometabolic health in young women with overweight: the NewStart randomised trial

**DOI:** 10.1017/S0007114524003015

**Published:** 2025-01-14

**Authors:** Line Barner Dalgaard, Line Thams, Jon Skovgaard Jensen, Astrid Ank Jørgensen, Andreas Breenfeldt Andersen, Kasper Degn Gejl, Hanne Christine Bertram, Mette Hansen

**Affiliations:** 1 Research Unit for Exercise Biology, Department of Public Health, Aarhus University, Aarhus, Denmark; 2 Department of Medicine, Gødstrup Hospital, Herning, Denmark; 3 Orthopaedic Research Unit, Department of Clinical Research, University of Southern Denmark, Odense, Denmark; 4 Department of Orthopaedics and Traumatoloy, Odense University Hospital, Odense, Denmark; 5 Research Unit of Muscle Physiology and Biomechanics, Department of Sports Science and Clinical Biomechanics, University of Southern Denmark, Odense, Denmark; 6 Department of Food Science, Aarhus University, Aarhus, Denmark

**Keywords:** Obesity, Dairy, Fat mass, Diet intervention, Satiety

## Abstract

The aim of this randomised controlled trial was to investigate the effects of breakfast high or low in protein on body composition and cardiometabolic markers in young women with overweight. In total, fifty-six women aged 18–30 years consumed a breakfast containing either high protein (34 g protein, *n* 26) or low protein (6 g protein, *n* 30) for 12 weeks. Measurements of body composition by dual-energy X-ray absorptiometry, waist circumference, glucose tolerance, fasting glucose, insulin and lipid profile were performed before and after this period. The primary outcome was fat mass. Satiety and hunger were evaluated by self-reported Visual Analogue Scale (VAS) scores. Dietary intake was estimated by 4-d dietary records, and calcium intake was estimated by FFQ. At baseline, relative daily protein intake was 15·2 ± 2·8 E%, which increased to 19·3 ± 3·4 E% in high protein but was unchanged in low protein (*P* < 0·001 between groups). High protein reported higher satiety compared with low protein (*P* = 0·02). Yet, no group differences were observed in changes in energy intake, body composition, blood lipid profile or measures of glucose tolerance (all *P* > 0·10). However, bone mineral content tended to increase in high protein (*P* = 0·05) and decrease in low protein (*P* = 0·07, interaction effect: *P* = 0·01). Conclusively, a high *v*. low content of protein in breakfast increased satiety but did not affect body composition or cardiometabolic markers in young women with overweight. This study adds to the sparse evidence on the effects of breakfast with different macronutrient compositions on health parameters in women with overweight. Registered at clinicaltrials.gov: NCT04518605.

Overweight and obesity are growing public health problems both worldwide and in Denmark, especially among women^(1–3)^. In Denmark, the prevalence of overweight, defined by having a BMI ≥ 25 kg/m^2^, has reached 40 % in women aged 25–34 years^(4)^. A high BMI in young women is associated with an increased risk for early heart failure and CVD^(5,6)^, which is the leading cause of death worldwide^(7)^, but also impaired glucose homeostasis^(8)^, leading to a higher risk of type 2 diabetes. Women within the age range of 20–45 years are at the highest risk of experiencing weight gain compared with younger and older women^(9)^, which based on recent characterisation of this group is likely linked to unhealthy lifestyle changes, for example, poor eating habits and inactive physical lifestyle^(10)^. Therefore, it is imperative to develop preventive strategies that promote a healthy body composition and cardiometabolic profile among young women, as even modest improvements may affect the long-term disease burden at the population level^(11)^.

Several cross-sectional studies report that eating breakfast is associated with a lower BMI and lower risk of type 2 diabetes and CVD compared with skipping breakfast^(12–17)^. However, longitudinal observational and randomised controlled studies have produced inconsistent findings^(18–20)^, possibly due to variations in the intervention breakfast. Further, in the general population, protein-rich diets have been shown to elicit favourable effects on body mass (BM) compared with normal-protein diets, but the effects on body composition and cardiometabolic health markers remain inconsistent^(21)^, potentially due to differences in study populations and the dietary protein source. Interestingly, intake of protein from dairy products has been shown to have stronger effects on insulin secretion and lower the risk of type 2 diabetes compared with protein from other food sources^(22,23)^. This may be attributed to the high content of leucine in dairy products, which has been shown to reduce fat mass (FM) and improve glucose tolerance in obese adults^(24)^. Additionally, a possible positive health effect of dairy product consumption may be partially explained by the accompanying increased intake of calcium and probiotic lactic acid bacteria. High calcium intake has been suggested to positively influence weight regulation^(25,26)^, while probiotics may enhance glycaemic control, reduce insulin resistance and improve lipid profiles^(27,28)^. In a randomised controlled study involving young women with overweight, we recently observed greater satiety and lower glucose response, although a comparable insulin response in the hours after a breakfast high in dairy protein to low in protein content^(29)^. Nevertheless, evidence on the long-term effects of protein-rich breakfasts on body composition and cardiometabolic health in young women with overweight and obesity is still lacking.

Therefore, we aimed to investigate the effects of consuming a dairy product-based, HP breakfast or an isoenergetic LP breakfast for 12 weeks on body composition and cardiometabolic markers in 18–30-year-old women with overweight or obesity. We hypothesised that the HP breakfast compared with the LP breakfast would increase satiety, reduce daily energy intake and thereby lower FM and waist circumference (WC), as well as improve glucose tolerance and blood lipid levels.

## Methods

### Study design and ethics

NewStart was a randomised controlled trial that included seventy-four Danish, women aged 18–30 years with overweight or obesity. They were randomly allocated to consume an isoenergetic high-protein (HP) or low-protein (LP) breakfast. The intervention lasted 12 weeks (range: 11–13 weeks). Participants were tested at baseline, mid-intervention and at the end of the intervention period (endpoint). The study was conducted between December 2019 and December 2021 at the Department of Public Health, Aarhus University, Denmark, and the Department of Sports Science and Clinical Biomechanics, University of Southern Denmark, Odense, Denmark. All procedures were conducted with the standards of the local ethical committee of the Central Denmark Region (MJ-1-10-72-220-19) and the Declaration of Helsinki. The trial was registered at clinicaltrials.gov with the ID: NCT04518605 before the recruitment was initiated. After trial registration, the protocol was modified due to restrictions due to the COVID-19 pandemic. Consequently, the planned exercise protocol was omitted, resulting in a nutritional intervention only, instead of a 2 × 2 factorial trial. As a result, an updated power calculation was performed, and enrolment was conducted accordingly. Nonetheless, the hypotheses were specified before the data were collected.

### Participants and recruitment

Women with overweight were recruited through advertisements in newspapers, the web page øwww.forsøgsperson.dk and through advertisements on social media. Women who responded to the invitation letter were pre-screened by telephone and were subsequently invited to an informational meeting. Written informed consent to participate was obtained from all participants.

Eligible women were 18–30 years old and had a BMI above 25 kg/m^2^. Exclusion criteria were (1) chronic diseases and/or use of prescription medication that could potentially affect BM or the study outcomes, (2) food allergies to any of the breakfast products and (3) exercising more than 2 h of cardio training per week and more than one strength training session per week.

**Figure 1.**
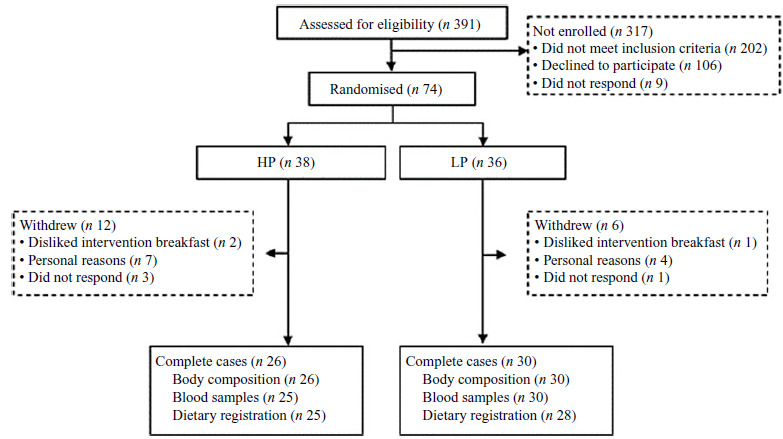
Flow chart. Complete cases refer to participants with both baseline and endpoint measurements. HP, high protein; LP, low protein.

A flow chart of the participants from recruitment to completion is shown in Fig. 1. Of the seventy-four randomised participants, eighteen withdrew during the study, mainly due to the COVID-19 pandemic. Participants with test data from both baseline and endpoint visits were included in the data analysis, resulting in a sample of *n* 56. Dietary registrations were incomplete for three participants, and blood samples were not obtained for one participant (Fig. 1).

### Randomisation and blinding

Block randomisation with twelve participants per block was used to allocate participants equally to the two intervention groups. An impartial staff member generated a computer-based randomisation list, from which sealed, identical envelopes containing the corresponding group allocations were produced. Participants randomly selected an envelope and were assigned to a group during the informational meeting. Due to the nature of the breakfast products, blinding of group allocation was not possible.

### Intervention

The nutritional compositions of the intervention breakfasts are shown in Table 1. Participants within each group received similar size breakfast meals. The isoenergetic HP and LP breakfasts contained 34 g and 6 g protein, respectively, while the carbohydrate content was only 35·9 g in the HP breakfast compared with 65·8 g in the LP breakfast, of which added sugar constituted 0 g in HP and 11·3 g in LP. Otherwise, the breakfasts were comparable in terms of energy density, fat content and dietary fibre. The HP breakfast consisted of 40 g oats and 300 g ‘skyr’, which is low-fat strained yogurt, similar to Greek yogurt. Skyr was provided by Arla Foods amba (Aarhus) and came in both unflavoured and fruit-flavoured variants in original packaging. The LP breakfast consisted of two slices of whole-grain toast bread, 20 g marmalade and 250 ml fruit juice. All breakfast products were commercially available in Denmark and provided free of charge to all participants. The breakfast substituted the participant’s habitual breakfast or lack thereof. Participants were instructed to record their breakfast intake daily on pre-coded recording sheets, which were used to assess compliance. Apart from the intervention meal, participants were encouraged to maintain their habitual dietary habits and participation in leisure-time physical activities during the study period. At the endpoint visit, participants were asked retrospectively to rate how much they liked the intervention breakfasts at week 2, mid-intervention and at endpoint, respectively. Additionally, at the endpoint visit, they evaluated their feeling of satiety and hunger after breakfast (AM), in the evening (PM) and overall, during the intervention period using VAS scores^(30)^. VAS scores were reported from 1 (‘not at all’ hungry/satisfied) to 10 (‘extremely’ hungry/satisfied).

**Table 1.** Nutritional composition of the intervention breakfasts*

	HP	LP
Energy (kcal)	322	331
Protein (g)	34·3	6·4
Carbohydrate (g)	35·9	65·8
Added sugar (g)	0·0	11·3
Fat (g)	3·4	3·5
Calcium (mg)	337	49
Fibre (g)	4·9	5·1

HP, high-protein breakfast; LP, low-protein breakfast.

* Data are presented as targeted daily intake.

### Anthropometry and body composition

The participants were not allowed to perform vigorous physical activity, take any medicine or drink alcohol 48 h prior to the test days. BM, height and WC were measured at baseline, mid-intervention and endpoint. The women were weighed once in the overnight fasted state in underwear on a Tanita BWB-800 digital scale. Standing height was measured to the nearest 0·1 cm with the head in the Frankfurt plane. BMI was calculated from height and BM, and the women were categorised as overweight (25·0–29·9 kg/m^2^) or obese (≥ 30 kg/m^2^) according to the cut-off points from the WHO cut-off points. WC was measured three times to the nearest 0·1 cm, midway between the lower rib and the iliac crest, on exhalation, while the women stood with the arms at their sides. The mean of all three measurements was used.

FM, lean mass (LM), visceral adipose tissue and markers of bone health were determined at baseline and endpoint via dual-energy X-ray absorptiometry scans using a GE Lunar Prodigy (GM Healthcare) with GE Healthcare software version 17, SP1^(31,32)^. The scans were conducted in the overnight fasted state after participants had emptied their bladder and while wearing metal-free underwear. Daily and weekly quality assurance tests of the equipment were performed.

### Glucose tolerance test and blood sampling

Venous blood samples were drawn from an antecubital vein following 10 min rest in a supine position. Overnight fasting blood samples were obtained at baseline, mid-intervention and endpoint to determine glucose, insulin, total cholesterol, HDL-cholesterol, LDL-cholesterol and TAG. Glucose tolerance was measured by an oral glucose tolerance test at baseline and endpoint (Hemocue Glucose 201 RT). Finger-stick blood samples were collected before, 30, 60, 90 and 120 min after consumption of 75 g glucose dissolved in 150 ml water. Participants rested in a quiet place during the test. Glucose tolerance was evaluated based on the glucose concentration measured (from finger prick) 2 h after the glucose bolus. Participants’ glucose tolerance was categorised as normal (< 7·8 mmol·l^−1^), pre-diabetic (7·8–11·1 mmol·l^−1^) or diabetic (> 11·1 mmol·l^−1^) using the WHO cut-points 30.

### Physical performance and activity level

Physical performance was measured through various tests at baseline. Maximal oxygen consumption (VO_2_max) was estimated using the Astrand fitness test^(33)^. The test was conducted on a bicycle ergometer (Monark, Ergomedic 828E). The cadence was maintained at 60 RPM throughout the test. After a 2 min warm-up, the bike’s resistance was adjusted in two steps to correspond with a stable heart rate of 120–140 BPM and 150–170 BPM, respectively (i.e. stabilisation after 3–4 min). VO_2_max (l·min^−1^) was then estimated by using the following equation:

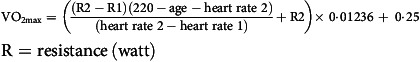




Hand grip strength was measured using a hand dynamometer with the grip adjusted according to hand size (SAEHAN SH5001). The test was performed with the dominant hand and the arm extended. Participants were instructed to squeeze as hard as possible for at least 3 s. The best performance (in kg) from three trials was used for analysis.

Countermovement jump was assessed using open hardware equipment and a contact platform (84·1 × 59·4 cm) from Chronojump (BoscoSystem). Participants stood straight with both feet on the platform at shoulder-width. Hands were placed on the hips, and the trunk was erect. Participants were instructed to jump vertically as high as possible by bending their knee to approximately 90° followed by full leg extension. The highest jump (in cm) from three trials was used for analysis.

Physical activity level (PAL) was evaluated via a self-reported questionnaire at baseline and endpoint. Participants were asked about frequency, intensity and time per d spent on physical activity and were asked to classify their daily living and physical demands at work on a scale from 1 to 4^(34)^. Total PAL was calculated using metabolic equivalent values according to accepted standards^(35)^.

### Dietary intake

Prior to the baseline, mid-intervention and endpoint visit, participants completed a weighed, 4-d dietary record using the web-based software Madlog (Madlog Aps, 2020) from which energy, macronutrient and dietary fibre intake were assessed. A study investigator reviewed the dietary records, and any abnormalities were clarified through dialogue during the upcoming examination visit. Calcium intake was estimated at baseline and endpoint using an electronic FFQ, referencing the previous month^(36)^.

### Statistical analyses

Descriptive data are presented as mean ± sd, median (25th–75th percentile) or *n* (%), as appropriate. All analyses were pre-specified and conducted using Stata/IC 17 and only participants with data from both baseline and endpoint for the specific outcome (complete cases). Statistical significance was set at *P*-values below 0·05, with trends towards significance at *P*-values below 0·1. The normal distribution of variables and model assumptions were evaluated visually using residual histograms and QQ plots. All models met the required model assumptions.

The sample size calculation of the NewStart study was based on the primary outcome, FM. The calculation used data from a previous study, where a 2 kg difference in FM was observed between a protein-rich breakfast group and the control group after 12 weeks^(37)^. Assuming that a similar difference in FM would be detected in the intervention groups in this trial, it was calculated that twenty-six women per group would be required to detect a group difference in FM of 2 kg after 12 weeks, with a statistical power of 80 %, a significance level of 5 % (two-sided) and a standard deviation (sd) of 2·5 kg. Given the uncertainty caused by COVID-19, seventy-four women were included, allowing for a potential 32 % dropout rate without compromising the statistical power.

Differences between non-completers and completers at baseline were tested using Pearson’s *χ* ^2^, Wilcoxon rank-sum or two-sample Student’s *t* test, as appropriate. Within-group changes from baseline to endpoint were tested with paired Student’s *t* test. Differences in dietary intake (energy intake, macronutrient intake, calcium) and PAL changes between the two study groups were analysed using one-way ANCOVA models, with the study group as a fixed factor, adjusted for baseline values. The effects of the breakfast intervention on the outcomes (BM, body composition, glucose tolerance measures and lipid profile) were evaluated as between-group post-intervention differences, adjusted for baseline values with two-way ANCOVA. To account for varying baseline BM, all analyses were re-run with BM included as covariate. Finally, per-protocol analyses were conducted, excluding participants who reported consuming fewer than six breakfasts per week.

## Results

### Subject characteristics

Of the seventy-four randomised women, a total of fifty-six were included in the final analyses (Fig. 1). The eighteen excluded women were mainly from the HP group (*n* 12) and tended to be younger (*P* = 0·06); however, they did not differ from the included women in terms of anthropometry or physical performance (*P* > 0·46, data not shown).

Table 2 presents baseline characteristics for the fifty-six completers. The included women were 25·1 ± 2·9 years old and classified as overweight (52%) or obese (48%). Most participants were non-smokers (89%) and did not engage in organised sports (80%). Their mean estimated fitness level was 30·4 ± 7·1 ml O_2_·kg^−1^·min^−1^. On average, the women regularly ate breakfast 4·8 ± 2·3 d per week. Habitual relative dietary protein intake was 15·2 ± 2·8 % of total energy (E%). Nearly one-third of participants (29%) had a fasting glucose level above 5·5 mmol·l^−1^, but below 7 mmol·l^−1^, classifying them as pre-diabetic.

**Table 2.** Baseline characteristics of included participants (*n* 56)* (Mean values and standard deviations; numbers and percentages)

*n*	HP		LP	
	26		30	
	Mean	sd	Mean	sd
Age (years)	25·3	2·7	24·8	3·0
Height (cm)	165·7	6·4	168·1	6·0
Body mass (kg)	83·7	13·5	87·8	16·9
BMI (kg/m^2^)	30·4	4·2	30·9	5·0
Weight category, *n* (%)†	*n*	%	*n*	%
Overweight	13	50 %	16	53 %
Obese	13	50 %	14	47 %
Habits				
Smoking‡; yes, *n* (%)	2	8 %	4	13 %
Organised exercise; yes, *n* (%)	6	24 %	5	17 %
	Mean	sd	Mean	sd
Breakfast (times per week)	5·3	2·1	4·4	2·4
Physical performance				
VO_2_max (ml·kg^−1^·min^−1^)§	27·2	6·2	33·1	6·8
Hand grip strength (kg)	33·0	8·2	36·1	13·5
Countermovement jump (cm)	16·0	3·9	16·4	v5·6

E%, energy percentage; HP, high protein; LP, low protein; VO_2_ max, maximal oxygen consumption.

* Values are mean, percentage ratios or sd.

† Defined as BMI above 25·0 and 30·0 kg/m^2^, respectively.

‡ Cigarettes and other nicotine products.

§ Estimated indirectly by the submaximal Astrand test.

### Randomisation and compliance

The randomisation was successful, though the LP group had a numerical but statistically insignificant higher fitness level compared with the HP group (33·1 *v*. 27·2 ml O_2_·kg^−1^·min^−1^, *P* = 0·02, respectively). The mean intervention duration was 12 weeks (range 11–13 weeks) with no significant difference between groups (*P* = 0·33). Median compliance to the breakfast meals was 96 % (IQR, 90–99 %) and appeared slightly higher in the LP group (98 %) compared with the HP group (94 %) (*P* = 0·06 between groups). In both groups, participants reported enjoying the intervention breakfast less after 12 weeks than after 2 weeks and 6 weeks, respectively (all *P* < 0·02, data not shown).

### Dietary intake and physical activity

Dietary intake is presented in Table 3. During the intervention period, total energy intake within the HP group decreased significantly compared with baseline (*P* = 0·02), but the change in energy intake did not differ significantly between groups (*P* = 0·18) (Table 3). The relative protein intake increased to 19·3 ± 3·4 E% in the HP group, while it remained stable in the LP group (*P* < 0·001 between groups). At baseline, there was no significant difference in protein intake (0·92 ± 0·22 g protein·kg^−1^·d^−1^ for HP and 0·82 ± 0·29 g protein·kg^−1^·d^−1^ for LP, *P* = 0·19). However, during the intervention, protein intake per kilogram per d was significantly higher in the HP group compared with the LP group (0·99 ± 0·32 g protein·kg^−1^·d^−1^ for HP and 0·69 ± 0·29 g protein·kg^−1^·d^−1^ for LP, *P* < 0·001).

**Table 3.** Dietary intake according to study group† (Mean values and standard deviations; 95 % confidence intervals)

		HP		LP		Δ Gr. Difference [95 % CI]
*n*		25		28		*P*-value‡
		Mean	sd	Mean	sd	
Energy intake (kJ·d^−1^)§	Baseline	8727	1598	8404	2147	
	Endpoint	7728	1876	8066	2060	–660 [–1635, 415]
	Change	–998*	1897	–338	1638	*P =* 0·18
Protein intake (E%)§	Baseline	15·2	3·0	15·1	2·7	
	Endpoint	19·3	3·4	14·3	1·9	4·9 [3·2, 6·7]
	Change	4·1*	3·7	–0·9	2·5	*P* < 0·001
Carbohydrate intake (E%)§	Baseline	48·2	7·6	48·5	5·9	
	Endpoint	46·2	4·6	53·8	7·1	–7·3 [–12·0, −2·6]
	Change	–2·0	7·5	5·3*	9·4	*P* < 0·001
Fat intake (E%)§	Baseline	35·2	7·6	36·4	5·3	
	Endpoint	33·0	5·7	33·5	7·4	0·7 [–3·8, 5·2]
	Change	–2·2	7·8	–2·9	8·5	*P =* 0·76
Fibre intake (g·d^−1^)§	Baseline	20·1	4·8	18·1	7·1	
	Endpoint	18·2	5·9	17·6	5·6	–1·5 [–5·1, 2·1]
	Change	–2·0	6·2	–0·5	6·8	*P =* 0·41
Calcium intake (mg·d^−1^)||	Baseline	597	538	518	433	
	Endpoint	742	486	353	198	310 [111, 509]
	Change	144	380	–165*	334	*P =* 0·003

E%, energy percentage; HP, high protein; LP, low protein.

*Indicates significant within-group changes (*P* < 0·05). Missing data from four subjects who did not complete the dietary registrations.

† Subjects with available data from both baseline and endpoint are included. Group values are presented as means and sd.

‡ *P*-values are the main effects of the intervention presented as estimated between-group differences (95 % CI) from two-way ANCOVA models adjusted for baseline. *P* < 0·05 was considered statistically significant.

§ Based on 4-d dietary records.

|| Based on FFQ.

In contrast, the relative carbohydrate intake increased in the LP group and remained unchanged in the HP group (*P* < 0·001 between groups). The estimated daily calcium intake was higher in the HP compared with the LP group (+386 mg·d^−1^, 95 % CI: +191, 580 mg·d^−1^, *P* < 0·001), due to a lowered calcium intake in LP (*P* = 0·01) and a trend towards increased calcium intake in HP (*P* = 0·08) during the intervention period. Self-reported PAL remained unchanged during the intervention compared with baseline in both groups and between groups (*P* = 0·98, data not shown).

### Intervention effects

**Figure 2.**
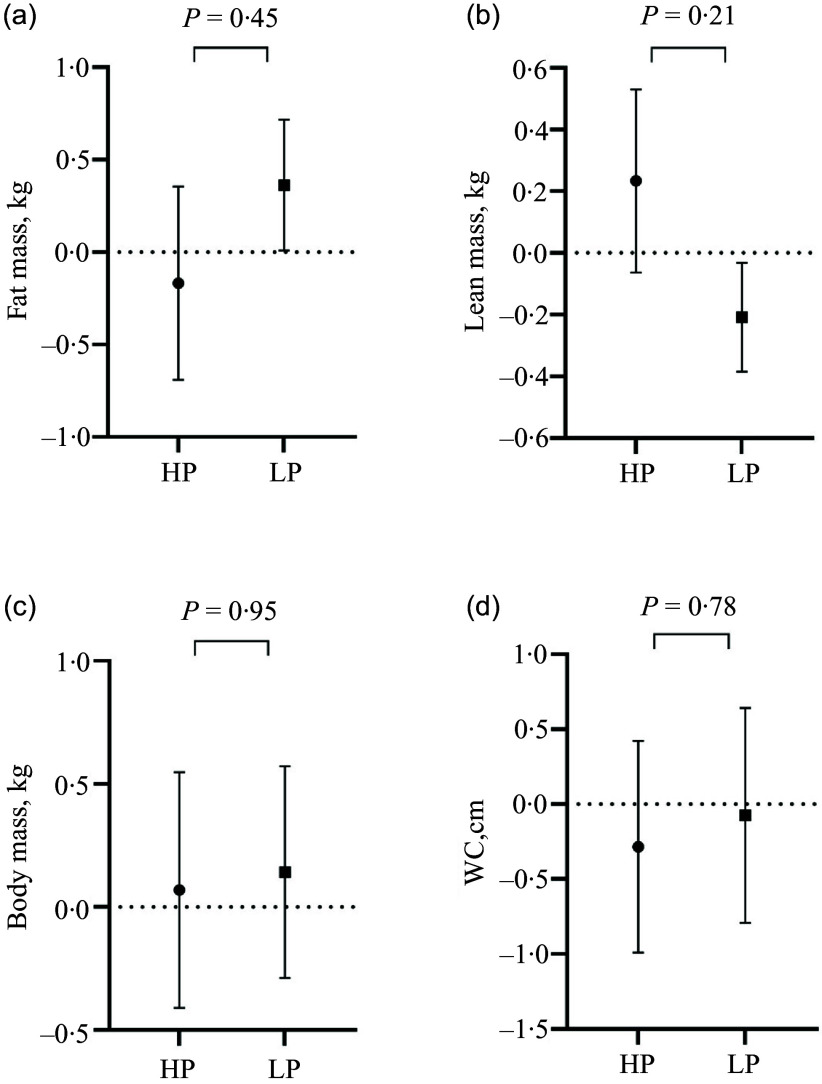
Mean (sem) changes in fat mass (a), lean mass (b), weight (c) and waist circumference (d) in the HP and LP group, as well as *P*-values for between-group differences. HP, high protein; LP, low protein; WC, waist circumference.

There were no effects of HP breakfast intake on FM, LM, BM or WC (Fig. 2). Additionally, visceral adipose tissue, BMI and the proportion of women classified as overweight/obese did not change during the intervention (data not shown). There were no within-group changes in any of these outcomes from baseline to endpoint (all *P* > 0·05). Covariate adjustments and per-protocol analyses did not alter the results (data not shown), nor did the exclusion of a few smokers result in any significant intervention effects on changes in BM, FM, blood lipids (total cholesterol; HDL-cholesterol, LDL-cholesterol) and fasting glucose (data not shown).

Bone mineral content (BMC) seemed to be influenced by the intervention (*β*: 20·1; 95 % CI: 4·4, 35·8 g; *P* = 0·01 between groups), due to a trend towards increased BMC in HP (*P* = 0·05) and a decreased BMC in LP (*P* = 0·07). However, bone mineral density, total bone mass and Z-score were not affected by the intervention.

Measures of glucose tolerance (fasting blood glucose, insulin, oral glucose tolerance test) and the blood lipid profile (HDL-cholesterol, LDL-cholesterol, TAG) did not show any significant between-group or within-group differences (Table 4), and adjusted analyses for baseline BM yielded similar results. However, the stratified explorative analyses of intervention effects in women with a BMI below 30 revealed a significant beneficial effect of HP *v.* LP on total cholesterol (–0·28 ± 0·32 mmol·l^−1^ *v.* 0·10 ± 0·42 mmol·l^−1^, *P* = 0·02) and LDL-cholesterol (–0·17 ± 0·31 *v.* 0·11 ± 0·34 mmol·l^−1^, *P* = 0·04). This effect is likely a direct effect of the change in macronutrient composition in the diet and not related to a differential effect on BM (*P* = 0·85) and FM (*P* = 0·67).

**Table 4.** Markers of cardiometabolic health in the study groups* (Mean values and standard deviations; 95 % confidence intervals)

		HP		LP		Δ Gr. Difference [95 % CI]
*n*		25		31		*P*-value†
		Mean	sd	Mean	sd	
Glucose (mmol·l^−1^)	Baseline	5·39	0·62	5·34	0·38	
	Endpoint	5·44	0·56	5·34	0·48	0·04 [–0·24, 0·31]
	Change	0·04	0·59	0·01	0·44	*P =* 0·79
Insulin (µmol·l^−1^)‡	Baseline	94·3	61·7	109·5	76·1	
	Endpoint	93·3	65·1	108·7	67·5	0·6 [–18·0, 19·2]
	Change	1·3	43·6	0·7	21·7	*P =* 0·95
Oral glucose tolerance (AUC)	Baseline	922	156	957	162	
	Endpoint	946	176	938	147	42 [–10, 94]
	Change	23	80	–19	107	*P =* 0·11
HOMA-IR‡	Baseline	3·82	2·56	4·46	3·49	
	Endpoint	3·76	2·69	4·43	3·25	–0·01 [–0·83, 0·81]
	Change	0·05	1·94	0·06	0·90	*P =* 0·98
Total C (mmol·l^−1^)	Baseline	4·65	0·95	4·66	0·74	
	Endpoint	4·55	1·08	4·63	0·73	–0·09 [–0·35, 0·18]
	Change	–0·10	0·48	–0·01	0·48	*P =* 0·52
HDL-cholesterol (mmol·l^−1^)	Baseline	1·35	0·32	1·39	0·29	
	Endpoint	1·33	0·29	1·35	0·26	0·02 [–0·07, 0·10]
	Change	–0·02	0·16	–0·04	0·14	*P =* 0·65
LDL-cholesterol (mmol·l^−1^)	Baseline	2·70	0·77	2·72	0·68	
	Endpoint	2·62	0·85	2·73	0·70	–0·10 [–0·31, 0·12]
	Change	–0·08	0·36	0·01	0·39	*P =* 0·37
TAG (mmol·l^−1^)‡	Baseline	1·26	0·80	1·21	0·49	
	Endpoint	1·22	0·85	1·22	0·46	–0·06 [–0·24, 0·13]
	Change	–0·04	0·31	0·01	0·37	*P =* 0·56

C, cholesterol; HOMA-IR, homeostasis model assessment of insulin resistance; HP, high protein; LP, low protein.

* Values are presented as mean and sd. All complete cases are included. Missing data from one subject in the HP group due to insufficient blood sampling.

† *P*-values are for the intervention effects, obtained from two-way ANCOVA models adjusted for baseline of the outcome. *P* < 0·05 was considered statistically significant.

‡ Insulin, HOMA-IR and TAG were log-transformed in the models, but the presented model estimates are back-transformed.

At baseline, one woman (HP group) was classified as diabetic (not prior diagnosed), and ten (18 %, four from HP, six from LP) was classified as pre-diabetic based on the oral glucose tolerance test. By the endpoint, an additional woman from LP was classified as diabetic and ten as pre-diabetic with no difference between groups (four from HP, six from LP). Women consuming the HP breakfast reported greater overall satiety compared with those consuming the LP breakfast (*P* = 0·02) (online Supplementary Fig. 1). Specifically, women in the HP group felt more satisfied (*P* < 0·001) and less hungry (*P* = 0·009) in the hours following the breakfast meal compared with women in the LP group, while evening satiety and hunger did not differ between groups (*P* > 0·51).

## Discussion

The present study showed no significant effects of consuming a protein-rich breakfast compared with the LP breakfast low on changes in energy intake, BM, body composition or cardiometabolic markers, despite women consuming the HP breakfast reporting higher general satiety compared with those consuming the LP breakfast. An interesting secondary finding was that HP compared with LP may positively influence BMC, possibly influenced by a higher calcium intake in the HP group than the LP group.

The beneficial effects of a protein-rich breakfast on satiety observed in this study align with a previous study, which reported that a single meal containing 30 g of protein – comparable to the HP breakfast of our study – improved postprandial fullness compared with isoenergetic meals with lower protein content (15–25 g)^(38)^. Additionally, our findings are consistent with previous studies in young women with overweight or obesity^(29,39)^, which demonstrated that a protein-rich breakfast (35 g) improved daily fullness compared with an isoenergetic breakfast lower in protein (6–13 g) or skipping breakfast. However, as in the present study, daily energy intake in these acute studies did not differ statistically between the HP and LP breakfast groups^(29,39)^. This may explain why neither BM nor body composition was affected by the present intervention. Another 12-week randomised controlled trial study showed that a hypoenergetic diet (–750 kcal·d^−1^) with 30 E% *v.* 18 E% protein content reduced FM to the same extent (–6·6 kg) in women with overweight and obesity^(37)^. However, in that study, the HP diet also improved satiety and preserved LM, which may be due to increased muscle protein synthesis and net protein turnover stimulated by the HP intake^(40,41)^. While the study above involved energy restriction, we aimed to investigate whether enhanced protein intake at breakfast could positively influence satiety and thus reduce the *ad libitum* daily energy intake and BM and improve body composition over time. In fact, the reported daily energy intake was significantly lower in HP during the intervention period compared with the habitual energy intake (∼–1 MJ·d^−1^), which theoretically would correspond to a net difference in FM of approximately –3 kg if sustained over 12 weeks. Nonetheless, the FM was only numerically reduced to a minor extent (–168 g), suggesting that the actual average daily energy deficit during the intervention was minimal. When dividing the data set into women with a BMI below 30 and those with a BMI above 30 in an exploratory analysis, the HP breakfast appeared more effective in preventing increases in BM and FM in women with a BMI above 30 compared with those with a BMI below 30. However, the differences between the intervention groups were not significant (*P* = 0·38 for BM and *P* = 0·28 for FM). In women with a BMI above 30, the LP group experienced a non-significant gain in BM (1·1 ± 2·2 kg, *P* = 0·12), while the HP group remained weight stable (0·2 ± 2·5 kg, *P* = 0·76). Similarly, for FM, the LP group showed a non-significant gain (1·1 ± 1·9 kg, *P* = 0·34), whereas the HP group remained stable (–0·1 ± 3·3 kg, *P* = 0·28). The numeric differences in FM and BM in the women with a BMI above 30 were coupled with a trend towards a differential beneficial effect of HP *v.* LP on fasting blood glucose levels (–0·18 ± 0·43 mmol·l^−1^ for HP *v.* 0·18 ± 0·50 mmol·l^−1^ for LP, *P* = 0·08). Although the study was not powered for these stratified analyses, the data suggest that a protein-rich diet, compared with a carbohydrate-rich diet, may help prevent further increases in BM and FM in women with obesity. In support of this, carbohydrate uptake in the brain and the subsequent increase in subjective fullness are impaired in obese individuals compared with those of normal weight^(42)^.

Although not statistically significant, the LM was numerically higher in HP at endpoint compared with baseline (+230 g) in contrast to LP (–209 g). This finding suggests that protein intake may positively impact LM. Still, within a normal range of recommended protein intake (10–20 E%)^(35)^, as ingested in the present study (19 *v.* 15 E% protein), a stimulating effect of resistance training may be needed to enhance LM significantly. A meta-analysis based on nineteen randomised controlled studies showed no significant effect on LM of a daily protein intake higher than 0·85 g protein·kg^−1^·d^−1^ compared with 0·8 g protein·kg^−1^·d^−1^, whereas in three studies including resistance training, a protein intake (> 0·85 g protein·kg^−1^·d^−1^) higher than recommended had a positive effect on LM gain^(43)^. Also, another meta-analysis including twenty-four randomised controlled studies showed that a higher (> 1 g protein·kg^−1^·d^−1^) *v.* lower (< 1 g protein·kg^−1^·d^−1^) protein intake helped maintain LM during weight loss^(44)^. Since the HP breakfast in the present study did not lead to a significant weight loss compared with the LP breakfast or included a training intervention, and the PAL was not enhanced during the intervention, it is not surprising that LM was not markedly changed at endpoint.

**Figure 3.**
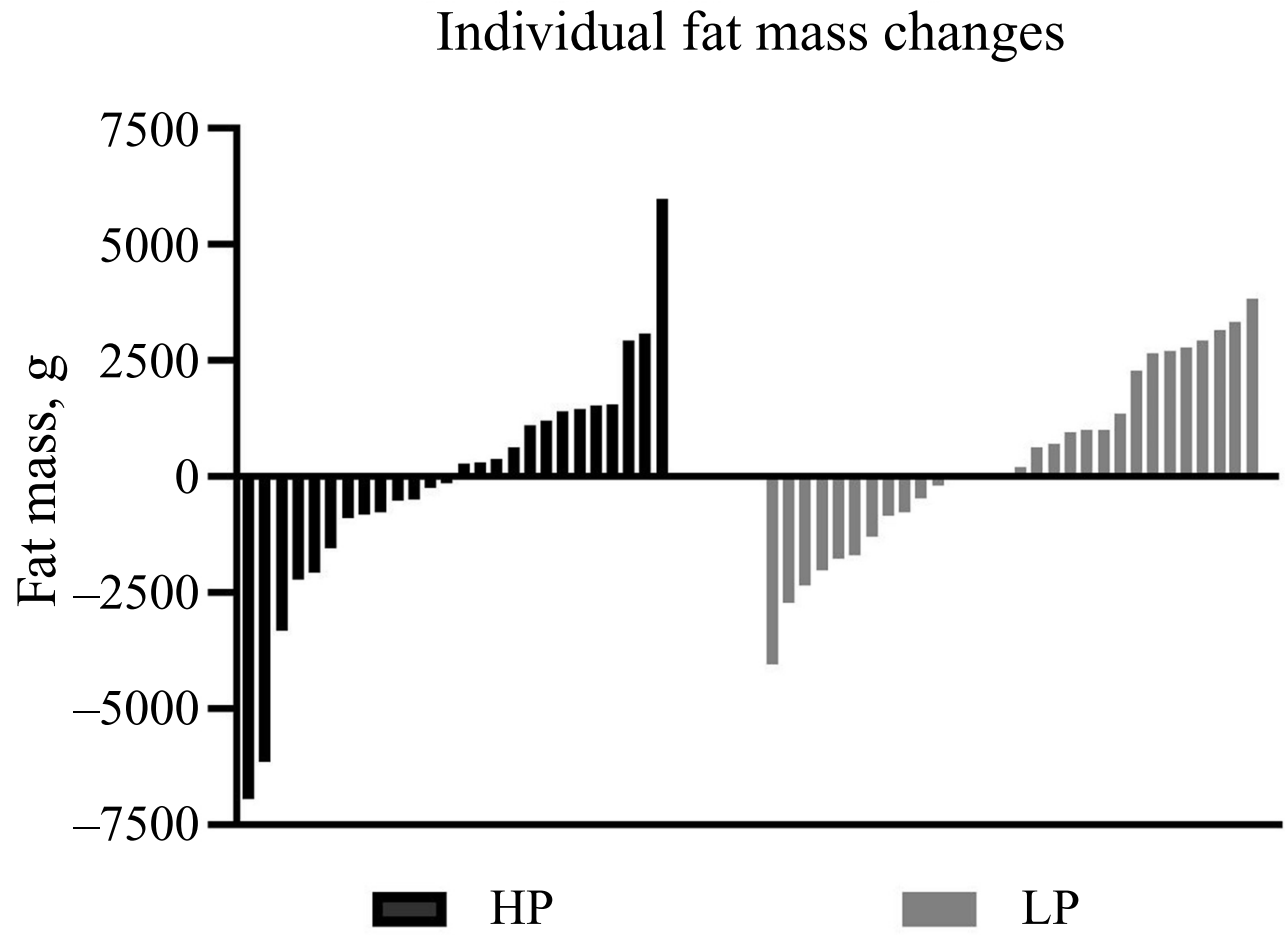
Individual changes in fat mass in the HP and LP group. HP, high protein; LP, low protein.

It is worth noting that we observed large inter-individual variations in changes in body composition in both groups during the intervention period (Fig. 3). FM changes ranged from −7·0 kg to +6·0 kg in HP and from −4·1 kg to +3·8 kg in LP. Similarly, LM changes varied significantly (–3·0 to 4·5 kg in HP; −1·8 to 1·8 kg in LP). This substantial range of body composition changes during the intervention period has also been observed in previous nutritional trials with adults who were overweight^(45)^, suggesting that ‘No single diet strategy fits all’.

Interestingly, BMC was increased in women consuming an HP breakfast compared with an LP breakfast over the 12-week intervention period. During this time, protein intake in the HP group increased significantly compared with baseline (76 ± 19 g protein·d^−1^ *v.* 85 ± 20 g protein·d^−1^, *P* = 0·002). In contrast, the LP group showed a tendency towards reduced protein intake (73 ± 17 g protein·d^−1^ *v.* 66 ± 15 g protein·d^−1^, *P* = 0·08). Additionally, relative protein intake in the LP group decreased significantly from 0·82 g protein·kg^−1^·d^−1^ to 0·69 ± 0·29 g protein·kg^−1^·d^−1^ (*P* = 0·02), falling below the recommendations of 0·83 g protein·kg^−1^·d^−1^. It is important to note that underreporting in food records is a common issue, and the protein intake recommendation does not account for excess FM. Nonetheless, these findings suggest that both absolute protein intake and changes in protein intake may impact BMC in young women. While the observed changes in BMC could be attributed to variations in protein intake between the intervention groups, they may also be a result of differences in calcium intake. At endpoint, the HP group consumed an average of 742 ± 486 mg calcium·d^−1^, which is close to the recommended intake of 800 mg·d^−1(35)^, whereas the calcium intake in the LP group decreased to 353 ± 198 mg·d^−1^. This also meant that the calcium:protein ratio differed significantly between the groups with a ratio of 31·9 mg calcium·g protein^−1^ for the HP group and a ratio of 22·8 mg calcium·g protein^−1^ for the LP group. To the best of our knowledge, no studies have thoroughly investigated the effect of calcium:protein ratio on bone health, but it has been suggested that a ratio of 30 mg calcium·g protein^−1^ is optimal^(46)^, which is supported by the present findings. Furthermore, our finding of higher BMC gain with HP is consistent with a previous randomised trial showing that diets higher in dairy product foods, dietary calcium and protein positively impacted key bone health biomarkers, such as bone turnover in premenopausal women with overweight and obesity^(25)^. Moreover, a meta-analysis of protein interventions in the general population showed that higher daily protein intake positively impacted bone mineral density compared with lower daily protein intake^(47)^. However, we observed no intervention effect on bone mineral density (*P* = 0·81), and it should be noted that the clinical relevance of our finding may be relatively low since the estimated difference between groups was small (∼20 g). We cannot exclude the possibility that the difference in BMC is influenced by measurement inaccuracies^(48)^. Therefore, future long-term studies should investigate the potential benefits of consuming foods with a combined high content of protein and calcium on bone health and on the risk of osteoporosis^(49)^.

The increased protein intake seemed not to affect any of the markers in the broad assessment of cardiometabolic health markers, despite all participants being classified as overweight or obese and the majority (95 %) being at increased risk for CVD based on WC (> 80 cm) at baseline. However, most women had normal blood lipid profiles at baseline. Therefore, it is not surprising that we did not observe any effects in these seemingly healthy measures during the relatively short 12-week intervention, which did not significantly alter BM or composition. Two previous trials have shown favourable effects of higher protein (30–31 E%) compared with moderate protein (16–18 E%) diets on TAG in women with overweight like those in the present study^(50,51)^. However, in both studies, the diets were hypoenergetic, and the positive change in TAG may have been due to greater weight loss^(51)^ or a higher fat-to-lean mass loss ratio^(50)^ with HP.

Several methodological considerations of the present study deserve mention. We aimed to explore a simple, easily applicable strategy to improve energy intake regulation by starting the day with a protein-rich breakfast, which is why the project was titled ‘NewStart’. We hypothesised that the HP breakfast would reduce the energy intake throughout the day, thereby positively influencing weight regulation. Strengths of our study include the use of dual-energy X-ray absorptiometry to assess body composition and bone health, as well as the inclusion of a broad panel of markers reflecting cardiometabolic health.

Although the dropout rate was relatively high, the women who completed the study demonstrated good compliance. Even though the intervention focused solely on the breakfast meal, the overall daily macronutrient intake was altered. The absolute and relative protein intake was significantly higher in the HP group compared with the LP group (19 *v.* 15 E% protein). However, a larger difference in protein intake between the groups might have resulted in more pronounced differences in the outcome parameters over time. Due to the nature of the breakfast products, we were unable to blind the intervention, but the investigators were re-blinded prior to data analysis. Since all participants were young women with sufficient protein intake and relatively healthy cardiometabolic markers, further studies are needed to explore the impact of breakfast composition on young women diagnosed with cardiometabolic diseases.

The use of oral contraceptives was not an exclusion criterion, enhancing the representativeness of the study population for this age group, as hormonal contraceptives were the most used contraception method at the time of data collection^(52)^. We did not track changes in hormonal contraceptive use during the study, but we assume that the distribution of contraceptive users and those who changed their contraceptive method during the intervention was balanced between the two randomised groups.

The intervention period lasted 12 weeks, equivalent to three oral contraceptive pill cycles or, on average, three menstrual cycles. Since the duration of the menstrual cycle can vary both within and between individuals, we cannot guarantee that all post-tests were conducted during the same menstrual phase as the pre-tests. However, on average, the post-tests were likely performed within the same menstrual cycle as the pre-tests.

Although some studies suggest that BM may fluctuate across the menstrual cycle, we aimed to assess the cumulative effect on body composition over a 3-month period, which should minimise the impact of minor day-to-day variations in BM at the group level.

### Conclusions

We found that consuming a high- *v.* low-protein breakfast for 12 weeks did not affect body composition or cardiometabolic health in young women with overweight. Although women consuming the HP breakfast reported higher satiety than women with the LP breakfast, daily energy intake was not significantly different between groups. However, a secondary finding of interest was that BMC increased in the group consuming a dairy product-based, protein-rich breakfast. More studies are needed to further our understanding of the potential positive health effects of eating a protein and calcium-rich breakfast in women at enhanced risk of cardiometabolic diseases.

## Supporting information

Dalgaard et al. supplementary material Dalgaard et al. supplementary material

## Supplementary material

For supplementary material/s referred to in this article, please visit https://doi.org/10.1017/S0007114524003015.
