# Supplementary figures and images for: No effects of high- v. low-protein breakfast on body composition and cardiometabolic health in young women with overweight: the NewStart randomised trial
Source: Br J Nutr. 2024 Nov 26;133(1):126–35. doi: 10.1017/S0007114524003015 (PMC11793950; doi:10.1017/S0007114524003015)

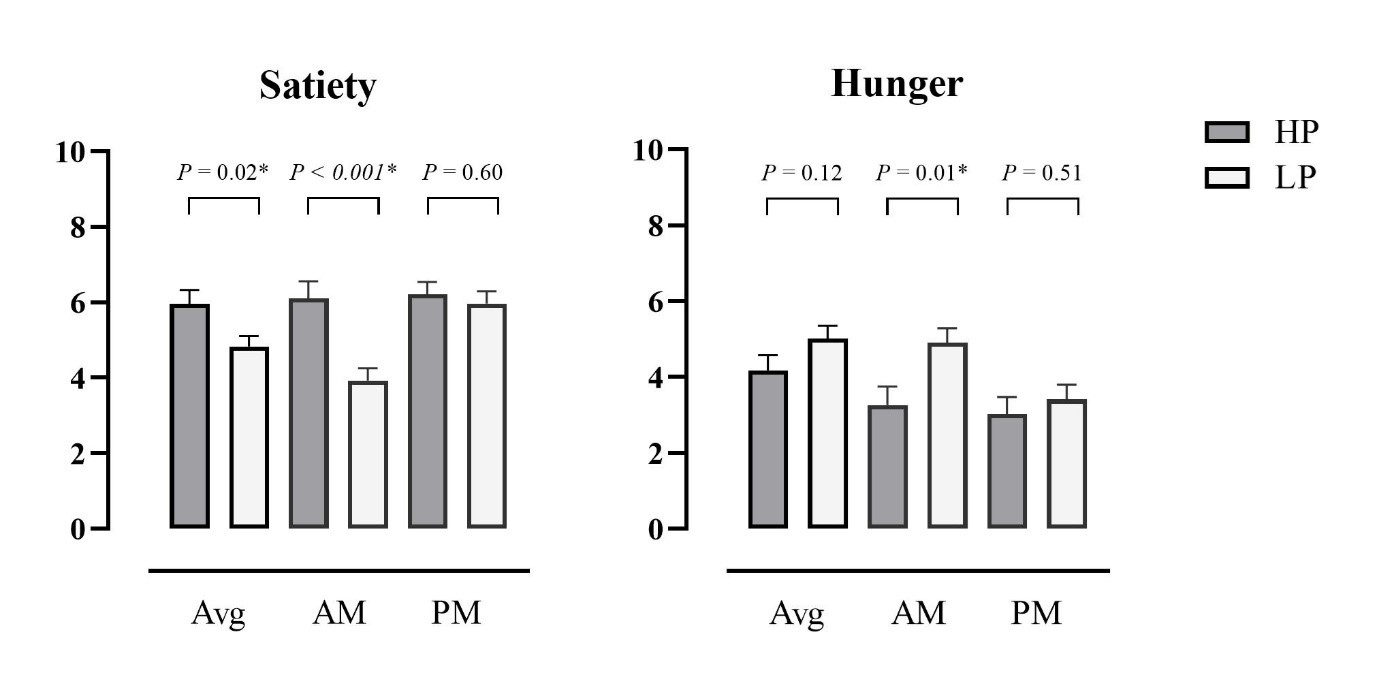

Supplement: Dalgaard et al. supplementary material [file S0007114524003015sup001.zip › SupFig1.jpg]
